# Undifferentiated Pleomorphic Sarcoma Presenting as Abdominal Pain with a Pulsatile Mass

**DOI:** 10.1155/2016/8251043

**Published:** 2016-08-03

**Authors:** Arash Moradi, Abolfazl Afsharfard, Khashayar Atqiaee

**Affiliations:** ^1^Department of General and Vascular Surgery, Shohada-e-Tajrish Medical Center, Shahid Beheshti University of Medical Sciences, Tehran, Iran; ^2^Department of Pediatric Surgery, Mofid Children's Hospital, Shahid Beheshti University of Medical Sciences, Tehran, Iran

## Abstract

Malignant fibrous histiocytoma (MFH) is a rare tumor that mostly involves adults aged 50 to 70. The most common anatomic location is the lower extremities. MFH of the retroperitoneum usually manifests late in its course and may be initially mistaken with other more common diagnosis. Here, the authors describe a 60-year-old man that was brought to the emergency department with a chief complaint of periumbilical abdominal pain. Our patient presented with symptoms consistent with a symptomatic aortic aneurysm, but a mass was encountered during surgery. In such circumstances the diagnosis of malignant sarcoma must be kept in mind and attempts at full resection with tumor-free margins are necessary.

## 1. Background

Malignant fibrous histiocytoma (MFH) was first described in 1961 by Kauffman and Stout as a histiocytic tumor with storiform growth in children [1]. Its malignant nature combined with an uncertainty regarding its origin and ability to involve both bone and soft tissue has given it the characteristics of a mysterious tumor, which is now accepted as a formal diagnosis of “undifferentiated pleomorphic sarcoma not otherwise specified” [2]. Various subtypes of MFH have been recognized including storiform-pleomorphic, myxoid, giant cell, and inflammatory [3]. Of these the storiform-pleomorphic subtype is the most common accounting for around 70% of the cases.

This rare tumor mostly involves adults aged 50 to 70, with a slight tendency towards male gender. The most common anatomic location is the lower extremities and the most common presentation is a growing painless soft tissue lump. Here we present the case of a 60-year-old man who came to the emergency department with a complaint of abdominal pain.

## 2. Case Report

A 60-year-old man was brought to the emergency department with a chief complaint of periumbilical abdominal pain. He suffered from abdominal pain for two weeks but it was significantly increased in the three days prior to admission. The pain radiated to the back and no accompanying signs or symptoms were present. It was described as a constant pain without any alleviating or exacerbating factors. The patient was a heavy smoker without any history of previous surgery or drug use.

The patient showed stable vital signs and a nuisance general appearance. No objective signs of weight loss or cachexia were observed. The physical exam was normal in the head and neck, chest, and extremities. In the abdomen a pulsatile mass was palpated in the periumbilical region. Routine laboratory tests revealed mild normocytic normochromic anemia, normal ESR and CRP, and liver function and diagnostic tests within normal limits.

**Figure 1:**
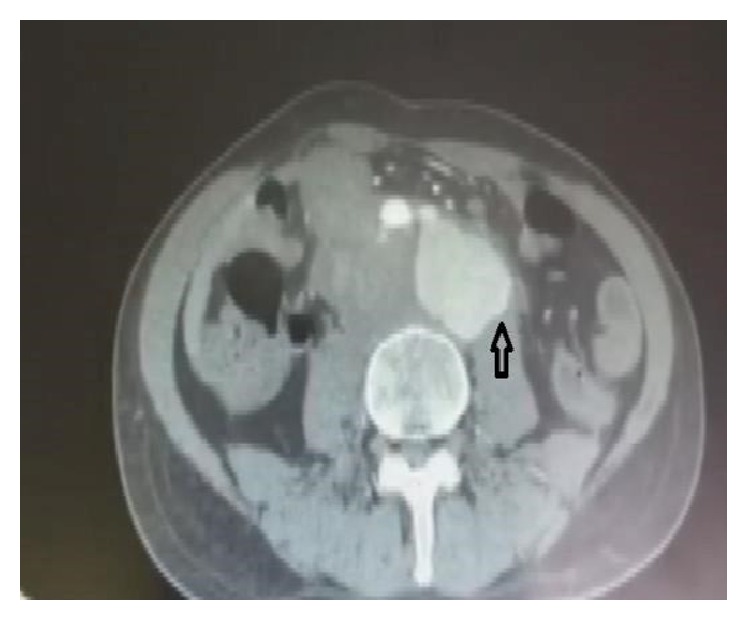
Large retroperitoneal mass.

Due to the pulsatile nature of the mass a CT angiography of the abdomen was ordered to look for vascular pathology. The tomography revealed a 5-by-4 cm pseudoaneurysm with hematoma formation in the infrarenal aorta alongside diffuse dilation of the infrarenal inferior vena cava with extension into both common iliac veins (Figure 1), and the patient was prepped for surgical repair.

During surgery a pulsatile mass 20 by 20 cm in diameter with surrounding hematoma was observed in left zones 1 and 2. Upon further investigation a huge retroperitoneal mass 11 by 8 by 6 cm in diameter with apparent local invasion to the aorta and the inferior vena cava was found. En bloc resection was attempted along with involved vascular segments. The abdominal aorta was reconstructed using 16 mm-by-8 mm Dacron bifurcated graft, while the IVC was ligated below the renal veins but perhaps because of chronic process of disease no lower extremity edema happened and the patient was discharged after a week.

**Figure 2:**
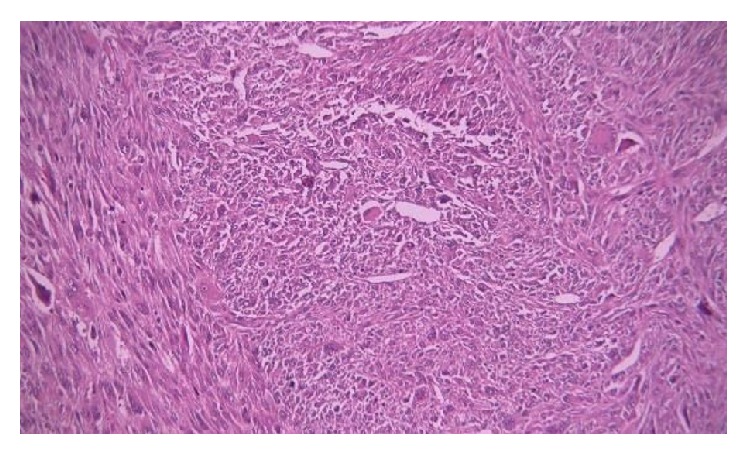
Hematoxylin and eosin stained section at 400x magnification demonstrating highly pleomorphic cells with prominent nucleoli in a storiform pattern.

Pathologic evaluation of the specimen showed an undifferentiated pleomorphic sarcoma (a.k.a. malignant fibrous histiocytoma) with a histologic grade according to French Federation of Cancer Centers Sarcoma Group III (tumor differentiation: III, mitotic rate >20/HPF: III, and tumor necrosis <50%: I) (Figure 2). There was no evidence of lymph-vascular or perineural invasion. Aneurysmal wall resection of the abdominal aorta with blood clot and atherosclerosis was also reported. The specimen was also positive for vimentin (patchy areas), SMA (small portions), and CD68 (most parts).

## 3. Discussion

Malignant fibrous histiocytoma is a sarcoma of mesenchymal origin affecting soft tissues of the body, particularly the extremities and retroperitoneum, yet it has been reported in almost all parts of the body [4–7]. The term malignant fibrous histiocytoma is believed to be a misnomer since the precise origin of MFH cells has been disputed and the concept of fibrohistiocytic differentiation has been challenged [8]. For this, the WHO has defined the tumor and its subtypes under undifferentiated pleomorphic sarcoma (UPS) not otherwise specified (NOS). Most undifferentiated high-grade pleomorphic sarcomas and undifferentiated pleomorphic sarcomas with giant cells occur in the deep soft tissues of the extremities while undifferentiated pleomorphic sarcoma with prominent inflammation is most commonly seen in the retroperitoneum [2].

Retroperitoneal and intra-abdominal tumors may present with constitutional symptoms, including fever, malaise, and weight loss [9, 10]. The tumor is often large at presentation since it usually goes unnoticed for a long time and may cause displacement of the bowel, kidney, ureter, and/or bladder. In our patient the tumor had displaced an abdominal aortic aneurysm anteriorly thus creating the unique clinical presentation of abdominal pain and pulsatile mass.

Although the best imaging modality for evaluation of the tumor is Magnetic Resonance Imaging (MRI), for intra-abdominal masses usually this is not the initial step. Generally, retroperitoneal undifferentiated pleomorphic sarcomas manifest as heterogeneous masses with areas of hemorrhage and necrosis and occasionally focal or diffuse coarse calcifications. While invasion of the abdominal musculature is a well-recognized phenomenon, the renal veins, or inferior vena cava, are not invaded [11, 12]. In our patient too despite the initial impression of vascular invasion in the operating room, pathologic reports confirmed that the vascular invasion had not occurred.

One must bear in mind that the pathological diagnosis of MFH/UPS is a diagnosis of exclusion. In order to rule out a pleomorphic nonmesenchymal neoplasm resembling a pleomorphic sarcoma, pleomorphic sarcoma as a result of dedifferentiation, and pleomorphic sarcoma with a specific line of differentiation, a combination of sampling and immunohistochemistry should be used.

Microscopically, UPS has a highly variable morphologic pattern and shows storiform to haphazardly arranged pleomorphic areas. In immunohistochemistry they show features of fibroblasts/myofibroblasts [8].

The current treatment of choice for primary MFH/UPS is wide surgical resection aiming at tumor-free margins. While adjuvant radiotherapy has become an integral part of treatment, the role of chemotherapy is still controversial in these tumors [13–15].

## 4. Conclusion

MFH/UPS of the retroperitoneum usually manifests late in its course and may be initially mistaken with other more common diagnoses. Our patient presented with symptoms consistent with a symptomatic aortic aneurysm, but a mass was encountered during surgery. In such circumstances the diagnosis of malignant sarcoma must be kept in mind and attempts at full resection with tumor-free margins are necessary.
